# Case Report of *Salmonella* and HHV-6 Meningitis in an Infant

**DOI:** 10.3390/pediatric17050094

**Published:** 2025-09-15

**Authors:** Sara Abed, Tahani Asiri, Razan Alzahrani, Wujud Hunjur

**Affiliations:** 1 Department of Pediatrics, King Abdullah Specialized Children’s Hospital, Ministry of National Guard Health Affairs (MNGHA), King Saud Bin Abdulaziz University for Health Sciences, King Abdullah International Medical Research Center, Jeddah 21423, Saudi Arabia; 2 Department of Pediatrics, King Abdullah Specialized Children’s Hospital, Ministry of National Guard Health Affairs (MNGHA), Jeddah 21423, Saudi Arabia; asirita1@mngha.med.sa (T.A.); hunjurwu@mngha.med.sa (W.H.); 3 East Jeddah Hospital, Jeddah 22253, Saudi Arabia; ralzahrani43@moh.gov.sa

**Keywords:** case report, *Salmonella* meningitis, human herpesvirus 6 meningitis

## Abstract

Bacterial meningitis is one of the most serious infections. *Salmonella* meningitis is associated with a high prevalence of long-term adverse outcomes, often linked to acute complications and a broad range of potential neurological sequelae following the infection. Acute complications such as brain abscesses and chronic complications such as hearing loss and developmental delay. In this report, we present a case of a 2-month-old male patient with seizures, hypoactivity and respiratory symptoms, who was found to have Salmonella bacteremia complicated by Salmonella and Human Herpes Virus-6 (HHV-6) meningitis, as well as rhinovirus bronchiolitis, along with follow-up findings. The patient’s data, including demographics, presenting symptoms, physical examination findings, and whole exome sequence results, as well as investigations such as complete blood count (CBC), cerebrospinal fluid (CSF) analysis, liver enzyme levels, and imaging findings, were collected from the electronic medical record system using a case report form. In addition, immunological workups were performed, as serious Salmonella infections were more common in immunocompromised patients. In the literature, there was no clear correlation between Salmonella and HHV-6 meningitis, rhinovirus bronchiolitis, and the complications that developed in this infant. This case report provides valuable insights into the clinical spectrum and long-term outcomes of patients with *Salmonella* meningitis.

## 1. Introduction

Bacterial meningitis is a severe, life-threatening central nervous system infection that requires immediate medical attention. Despite appropriate treatment, it is associated with high morbidity and mortality [1]. *Salmonella* meningitis, caused by a Gram-negative bacillus from the Enterobacteriaceae family, is uncommon. However, it is most frequently diagnosed in infants under 1 year of age, especially those younger than 3 months, in developing countries with tropical climates [2]. *Salmonella* meningitis can manifest with nonspecific symptoms, including fever, irritability, lethargy, and altered mental status, which can make diagnosis challenging. Confirmatory diagnosis requires the isolation of the organism from either cerebrospinal fluid (CSF) or blood cultures. *Salmonella* meningitis is associated with a high prevalence of morbidity (50–90%), presenting with various complications, and a high mortality rate of up to 50–70% [3]. Acute-phase complications of *Salmonella* meningitis may lead to subsequent motor disabilities, epilepsy, language delay, intellectual impairment, as well as visual and hearing impairments [3]. The first case of *Salmonella* meningitis was reported by Gohn in 1907, while Burrows first reported brain abscesses due to Salmonella in 1959 [4]. A retrospective study conducted in Saudi Arabia from 1999 to 2016 evaluated the neurological complications and long-term outcomes of 14 children aged 14 years or younger who were diagnosed with Salmonella meningitis at King Khalid University Hospital. The study showed that the majority of patients, 12 (86%), developed acute neurologic complications, including subdural empyema and multiple cerebral infarcts, 8 (57%), hydrocephalus, 5 (36%), ventriculitis, 4 (29%), and cerebral venous sinus thrombosis, 3 (21%). A total of 4 (28.5%) died due to complications of Salmonella meningitis. Among the survivors, four achieved full recovery, while six (60%) experienced long-term neurologic complications, including hydrocephalus, cerebral palsy, developmental delay, and epilepsy in five, four, three, and three patients, respectively [5]. While viruses are the leading cause of meningitis in children, little is known about long-term outcomes after hospital discharge. A systematic review examined studies reporting post-discharge outcomes in children under 16 with viral meningitis. Four studies looked at non-enterovirus 71 meningitis. A U.S. study noted minor language delays at the three-year mark, while an Australian study found no language issues. A Fijian study reported hearing loss in 2 of 8 children, unlike a large UK study that found none among 668 infants [6]. The involvement of human herpesviruses 6 and 7 (HHV-6 and HHV-7) in central nervous system (CNS) conditions remains an area of ongoing investigation. These viruses have been associated with a broad spectrum of neurological issues, ranging from asymptomatic infections and febrile seizures to meningitis and fatal encephalitis. HHV-6 DNA has been identified in CSF and other fluids through polymerase chain reaction (PCR). In one study, PCR testing of CSF from 245 patients revealed HHV-6 DNA in three cases, all of which were in infants younger than two months [7]. A study investigated the long-term outcomes of HHV-6 meningoencephalitis in previously healthy children. Over a decade-long follow-up, three out of 210 pediatric cases were documented. Two of them experienced significant neurological impairments, one with persistent speech difficulties and the other with lasting hemiplegia and bilateral vision loss. These findings highlight the potential for both severe and mild disease courses, underscoring the importance of careful follow-up [8].

Rhinovirus is a common respiratory pathogen in children. Central nervous system involvement is extremely rare, although a single case was reported in 2019. A previously healthy 2-year-old Japanese boy developed a fever, followed by seizures and lethargy. His cerebrospinal fluid cell count and protein level were slightly increased; brain magnetic resonance imaging showed abnormal intensities in the bilateral cerebellar dentate nuclei, which were prominent in diffusion-weighted images. After his consciousness disturbance improved, cerebellar dysfunction became apparent. He was treated symptomatically without steroids or any other immunosuppressants. He almost recovered within a few months; however, cerebellar atrophy became evident on brain magnetic resonance imaging. Using acute specimens, human rhinovirus A was detected in his throat swab and cerebrospinal fluid [9].

In this report, we present a case of a 2-month-old male patient with Salmonella bacteremia and meningitis, concurrent with HHV-6 meningitis, and provide long-term follow-up.

## 2. Case Presentation

A 2-month-old male infant presented to the emergency department with a history of fever, cough, and abnormal movements. The patient was born preterm at 36 weeks and 5 days via spontaneous vaginal delivery, with Apgar scores of 9 at both 1 and 5 min. His birth weight was 3 kg, and his head circumference was 34 cm. Neonatal intensive care was not required; he passed newborn screening for critical congenital heart defect and metabolic disease, and he received the birth vaccine (which is the first dose of the hepatitis B vaccine) before discharge. He was booked for a hearing screen test, which had not been performed yet at the time of presentation to the emergency department. The patient had no significant medical or surgical history and no known allergies. Developmentally, he did not fixate on or follow objects and exhibited a head lag; he was on formula feeding. The patient’s family lives in a small village in western Saudi Arabia and has a history of consanguinity. He has nine healthy siblings.

The infant presented with a fever, reaching 39 °C, that did not respond to antipyretics, accompanied by hypoactivity for 2 days, as well as cough and runny nose for 1 day. A positive history of choking episodes was reported both at home and in the emergency department. In addition, the patient experienced two episodes of apnea with cyanosis and two brief abnormal movements in the form of tonic–clonic seizures with eye-rolling, which were briefly controlled with lorazepam. The patient was initially admitted to the pediatric intensive care unit (PICU) on 23 September 2022. On initial physical examination, the patient was febrile, in respiratory distress, and dehydrated. Neurological examination showed an open, flat anterior fontanelle, bilaterally reactive pupils, and axial hypotonia.

Laboratory tests at admission revealed hyperinflammation, with elevated C-reactive protein (CRP) at 331.0 mg/L (reference value < 5 mg/L), procalcitonin at 130 mg/L, and leukopenia at 2.1 × 10^9^/L. The laboratory findings and progression are presented in Table 1 and Table 2.

The chest X-ray showed bilateral lower lobe opacities that could be consolidation versus atelectasis.

Brain magnetic resonance imaging (MRI) findings on 24 September 2022, were indicative of a hypoxic–ischemic insult, demonstrating hypoperfusion in the watershed areas of the periventricular white matter. Meningeal enhancement was consistent with meningitis, accompanied by bilateral basal ganglia and thalamic infarctions. The possibility of underlying abscesses or cerebritis could not be excluded.

The parents appeared understandably anxious and fearful following their son’s diagnosis of meningitis and hospitalization. The patient was in respiratory distress during the PICU stay, and a nasopharyngeal PCR swab tested positive for human rhinovirus/enterovirus. The patient was managed with nasal continuous positive airway pressure and nasogastric tube feeding. Antibiotics were initiated with Meropenem 40 mg/kg/dose three times daily for 3 days. After the culture results came back sensitive to ceftriaxone, the antibiotic was downgraded to ceftriaxone. Still, the patient’s fever spiked again on the second day of ceftriaxone initiation, and the inflammatory markers trended upward; therefore, the patient was shifted back to meropenem and continued on it. Furthermore, PCR of the second LP showed HHV-6, which was not detected in the first LP, indicating a primary HHV-6 infection. Given that he had complicated Salmonella meningitis, he was suspected of being immunodeficient, so he was started on ganciclovir 5 mg/kg/dose twice daily, too. The patient was transferred to the general ward on 4 October 2022, for continued care after he was stabilized.

A follow-up MRI on 9 October 2022, 2 weeks later, showed new subdural empyemas and worsening meningitis. The small abscess-like lesions near the ventricles had grown slightly compared to the earlier scan. Signs of previous hypoxic–ischemic injury were observed, with some improvement, including reduced swelling and improved blood flow in the periventricular white matter and posterior parietal lobes. The basal ganglia lesions could represent small abscesses, brain infection (cerebritis), or subacute strokes. The neurosurgical team determined that the patient did not require surgical intervention, as the empyema was not causing a mass effect and the patient remained clinically stable. The patient was continued on meropenem for 6 weeks, as a second MRI and after 2 weeks of proper treatment showed new collections, while ganciclovir was discontinued after the patient received 21 days of treatment.

**Figure 1:**
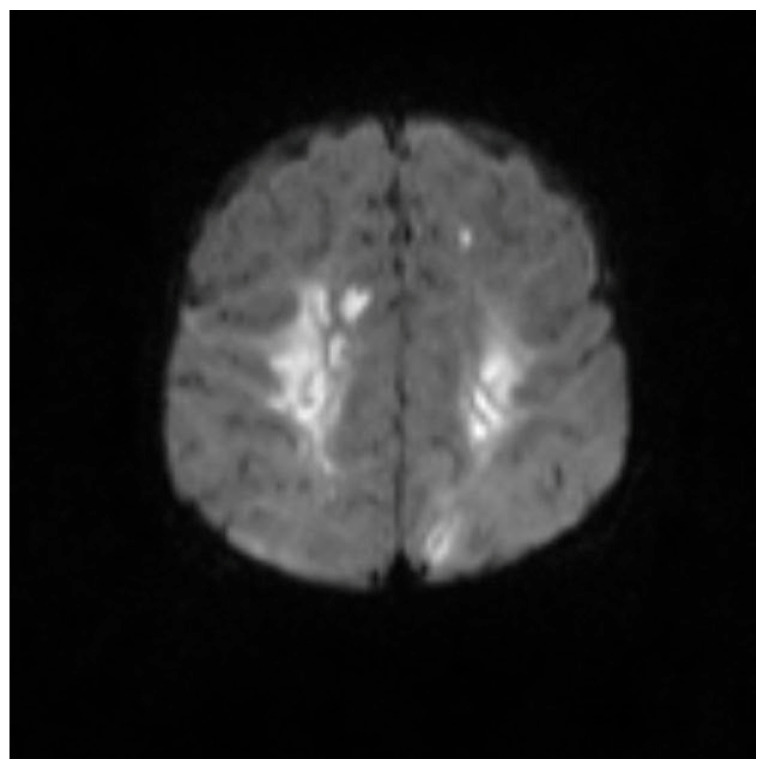
MRI brain scan showing the watershed area infarction.

**Figure 2:**
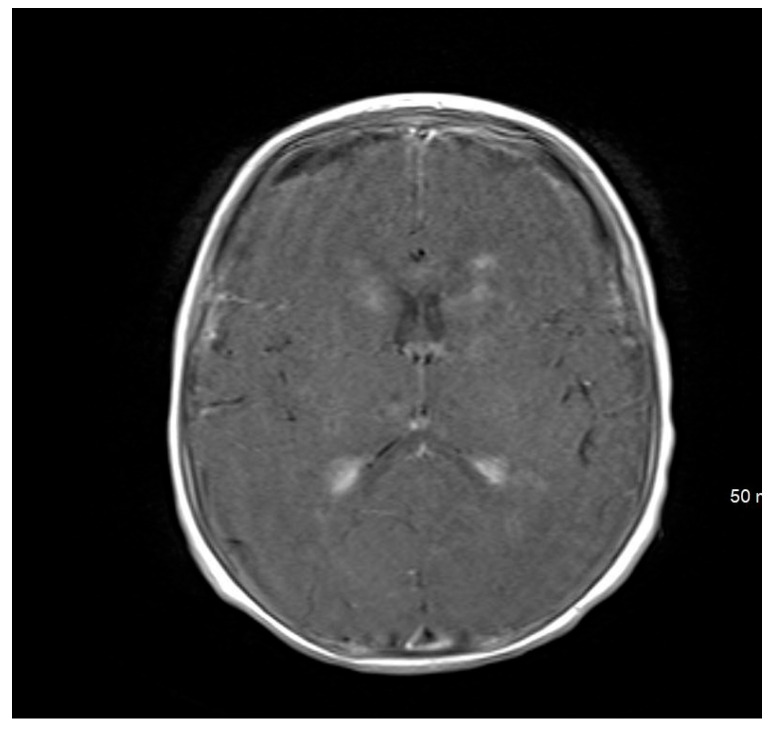
MRI brain scan showing meningeal enhancement.

**Figure 3:**
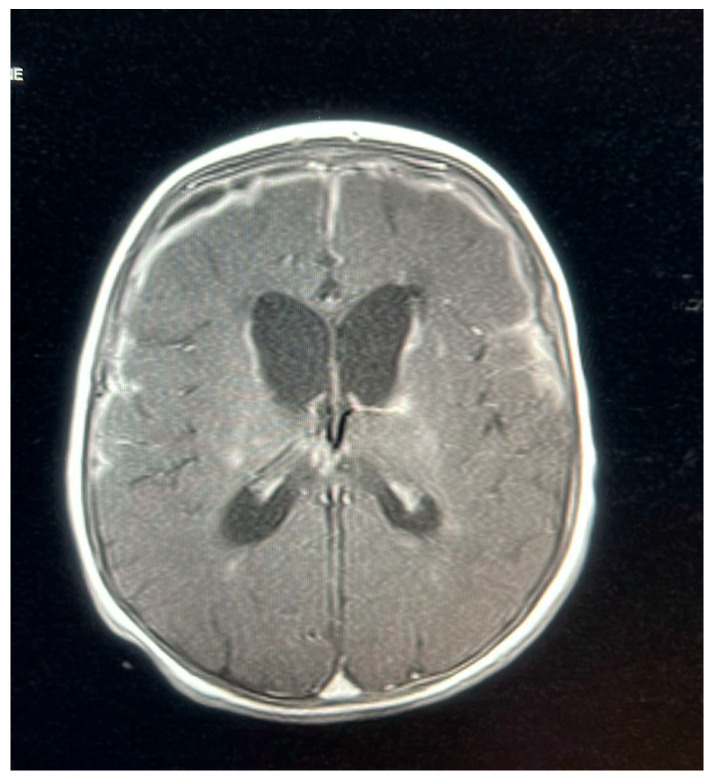
MRI brain scan showing worsening of leptomeningeal enhancement.

**Figure 4:**
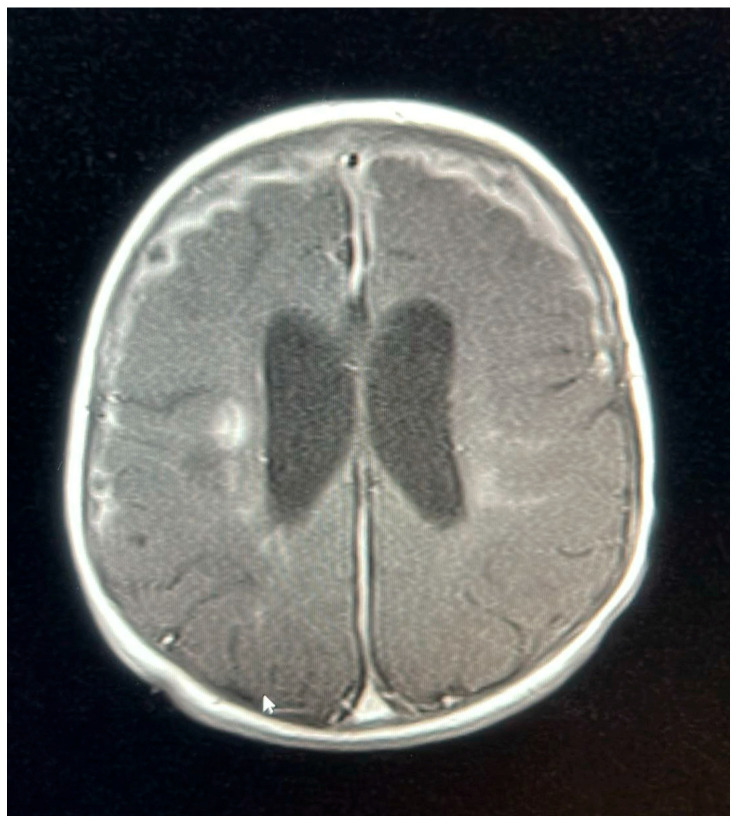
MRI brain scan showingsubdural empyema.

**Figure 5:**
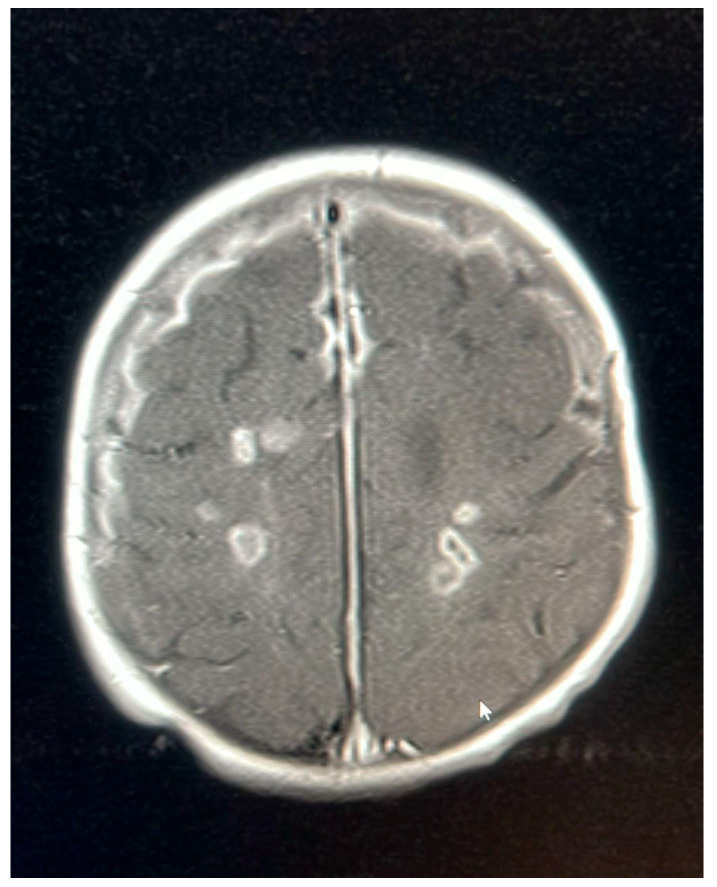
MRI brain scan showing periventricular lesions.

An MRI repeated on 11 November 2022, after 7 weeks of treatment, showed interval resolution of the abscesses and subdural empyema, with resolution of leptomeningeal disease. Expected changes were observed, including brain volume loss and periventricular leukomalacia, with the previously described foci of enhancement. The internal auditory canals appeared unremarkable (Figure 1, Figure 2, Figure 3, Figure 4 and Figure 5).

The patient experienced multiple seizures during the hospital stay. Electroencephalography (EEG) on 26 September 2022, revealed electrographic and electroclinical seizures originating from the right centrotemporal region. The patient was started on levetiracetam 15 mg/kg/dose twice daily, and phenobarbital 2 mg/kg/dose twice daily was added. He was discharged on these two antiepileptic medications. Whole-exome sequencing (WES) and whole-genome sequencing (WGS) were unremarkable, except for a secondary finding of a heterozygous mutation in the purine nucleoside phosphorylase (PNP) gene, which classified the individual as a carrier for a biotinidase (BTD) mutation in the heterozygous state. The family sought clear explanations regarding the expected outcomes and valued regular updates from the medical team.

After discharge on 23 November 2022, the patient attended multiple follow-up visits with various subspecialties. A hearing assessment revealed severe to profound sensorineural hearing loss in the left ear, while hearing in the right ear was normal. Focal seizures persisted for some time after discharge. Still, they were eventually controlled with three antiepileptic drugs (AEDs): Levetiracetam 15 mg/kg/dose twice daily, Phenobarbital 2 mg/kg/dose twice daily, and Vigabatrin 500 mg in the morning and 1000 mg at night. An eye examination at 9 months of age showed that the patient could fixate on and follow objects, with findings of a healthy disk, a flat retina, and myopic astigmatism. With further follow-up in neurology, the patient was noted to exhibit global developmental delays and severe spasticity, which required management with baclofen and physiotherapy. Additionally, the patient underwent an evaluation in the immunology clinic for an immune deficiency workup to rule out a potential immunodeficiency disorder associated with the invasive bacterial infection. Testing, including lymphocyte subset analysis, quantitative immunoglobulin levels, antibody titers for tetanus and diphtheria after vaccination, and human immunodeficiency virus (HIV) antigen tests, yielded unremarkable results. However, the oxidative burst assay for chronic granulomatous disease was not applicable as it is not available in the hospital.

## 3. Discussion

Bacterial meningitis is a medical emergency requiring immediate intervention to prevent the disease from progressing to serious complications, such as empyema, ventriculitis, and intracranial hemorrhage [10]. While bacterial and viral infections are the most common causes of meningitis, injuries, cancer, certain drugs, and other types of infections can also lead to meningitis. Notably, although not specific, elevated inflammatory markers, such as an increased Procalcitonin level, can provide supportive evidence in patients with *Salmonella* meningitis. In this patient, CSF analysis revealed a total leukocyte count of 336/mm^3^, with 10% neutrophils and 90% lymphocytes. The patient also demonstrated a high CRP level of 331 mg/dL and an increased serum Procalcitonin level (130 mg/dL).

A retrospective review of patients with spontaneous *Salmonella* meningitis from 1982 to 1994 evaluated school-age survivors based on developmental milestones reported by their parents and detailed neurological evaluations, including assessments of intelligence, hearing, visual, speech, and language. Of the 24 patients, seizures were noted in 15 (63%) before admission and 13 (54%) during hospitalization. Acute complications included hydrocephalus (50%), subdural collections (42%), cerebral infarction (33%), ventriculitis (25%), empyema (13%), intracranial abscess (8%), and cranial nerve palsy (8%). Three patients (13%) died during the acute phase of *Salmonella* meningitis. The 21 survivors, who were followed up at school age, exhibited various sequelae, including language disorders (52%), motor disabilities (48%), intelligence quotient < 80 (43%), epilepsy (33%), sensorineural hearing loss (17%), visual deficits (10%), abducens nerve palsy (5%), microcephaly (5%), and hydrocephalus (5%). Overall, among the 21 survivors, 6 (28.6%) had a good outcome, while mild, moderate, and severe sequelae were noted in 3 (14.2%), 6 (28.6%), and 6 (28.6%) patients, respectively [3].

*Salmonella* meningitis is associated with a high prevalence of long-term adverse outcomes, often linked to acute complications and a broad range of potential neurological sequelae. In our case, seizures during admission were initially difficult to control, requiring multiple adjustments in AEDs and dosage until the infection was fully resolved. Following the resolution, AEDs were gradually tapered. Moreover, the patient was found to have severe to profound sensorineural hearing loss in the left ear and developmental delay, both of which were recognized during scheduled follow-ups after discharge. On the other hand, a case report was published in 2024. A 2-month-old male infant reported Salmonella meningitis symptoms, such as fever, irritability, altered sensorium, and diarrhea. Clinical examination revealed bulging anterior fontanelles, dehydration, and sunken eyes. Screening for normal hearing, cranial ultrasound, and magnetic resonance imaging (MRI) showed no abnormalities in the brain. A cerebrospinal fluid (CSF) culture revealed Gram-negative Salmonella enterica bacilli. Treatment with meropenem and ampicillin was initiated after antibiotic susceptibility testing showed sensitivity. The patient’s cerebrospinal fluid parameters and bacterial growth improved after antibiotic therapy. Two weeks later, the baby was neurologically healthy and discharged [11].

*Salmonella* meningitis has a 70% higher likelihood of occurring in immunocompromised individuals [12]. Therefore, an immunological and genetic workup was conducted for this patient, yielding normal results. While viral infections are the most common etiologies of meningitis, the incidence of bacterial meningitis has significantly decreased due to vaccination. However, simultaneous bacterial and viral meningitis is uncommon. Furthermore, the medical literature has not clearly described a combined infection involving Salmonella and human herpesvirus-6 (HHV-6). HHV-6 can infect the central nervous system in both immunocompromised and immunocompetent patients [13]. In a cross-sectional study of 100 children under 2 years of age with febrile convulsions, HHV-6 was detected in CSF in 6% of the patients. Thus, primary infection with HHV-6 is associated with febrile convulsions, which puts patients at a higher risk of developing epilepsy, as demonstrated in our case [14]. Furthermore, a cohort study published by the American Academy of Pediatrics investigated the association between viral bronchiolitis in children under two years of age and concurrent serious bacterial infections, including bacteremia, meningitis, urinary tract infections, and pneumonia. The overall incidence of concurrent viral bronchiolitis and serious bacterial infections was low, at 4.2%. Among these, bacterial pneumonia presented the highest incidence, while bacterial meningitis was notably rare, comprising only 0.02% of cases [15]. Additionally, the existing literature highlights a lack of research addressing the connection between rhinovirus-induced bronchiolitis and the risk of developing bacterial meningitis.

## 4. Conclusions

The present study elucidates the clinical spectrum and long-term outcomes associated with Salmonella meningitis in affected patients. Infants, particularly those at an early age, who develop complicated meningitis are at an increased risk for significant sequelae, including sensorineural hearing loss, intractable seizures, and global developmental delays. Pediatricians should be aware of the potential for meningitis caused by Salmonella in children, as these complications also strain healthcare resources and governmental support systems. Early recognition of Salmonella infection and prompt therapeutic interventions may mitigate progression, potentially reducing the overall burden on patients, caregivers, and healthcare institutions.

## Figures and Tables

**Table 1:** Laboratory evaluation of the patient upon admission and discharge.

	Upon Admission	Upon Discharge	Normal Range and Unit
Hemoglobin	9.7	11.7	12.0–16.0 g/dL
MCV	92	86	80–100 fl
MCH	31	28	27–32 pg
White blood cell	2.1	4.9	4.0–11.0 × 10^9^/L
Lymphocyte count	1.03	3.35	1.0–4.0 × 10^9^/L
Neutrophil count	0.95	0.79	2.0–7.5 × 10^9^/L
Monocyte count	0.12	0.52	0.2–0.8 × 10^9^/L
Eosinophil count	0.01	3.35	0.0–0.5 × 10^9^/L
Platelet count	217	300	150–450 × 10^9^/L
Creatinine	34	22	60–110 mmol/L
Blood urea nitrogen	4.5	2.4	3.2–7.1 mmol/L
AST	38	42	10–40 U/L
ALT	28	29	7–56 U/L
C-reactive protein	331.6	16	<5 mg/L
Procalcitonin	130	0.09	<0.5 mg/L
Blood culture	*Salmonella* species, sensitive to gentamicin, ciprofloxacin, ceftriaxone, and ampicillin	No growth	No growth

ALT, alanine aminotransferase; AST, aspartate aminotransferase; MCV, mean corpuscular volume.

**Table 2:** Findings of CSF analysis.

CSF Investigations	Upon Admission	Repeated After 7 Days from Antibiotic Initiation	Repeated After 17 Days from Antibiotic Initiation	Normal Reference Values
Specimen Description	Turbid	Xanthochromic	Slightly turbid	Clear and colorless
CSF culture	Heavy growth, *Salmonella* species, sensitive to gentamicin, ciprofloxacin, ceftriaxone, and ampicillin	*Salmonella* species from broth only, sensitive to gentamicin, ciprofloxacin, ceftriaxone, and ampicillin	No growth	No growth
White blood cell	336 per CMM	336 per CMM	210 per CMM	0–5 per CM
Differential count	Mononuclear cells: 90% Polymorphonuclear: 10%	Mononuclear cells: 90% Polymorphonuclear: 10%	Mononuclear cells: 95% Polymorphonuclear: 5%	Lymphocytes 60–80%, Monocytes 15–45%, Neutrophils 0–6%
RBC	5 per CMM	10 per CMM	8 per CMM	0 per CMM
CSF protein	4.80 mg/mL	3.47 mg/mL	2.15 mg/mL	0.15–0.45 mg/mL
CSF glucose	<0.1 mmol/L	0.7 mmol/L	1.9 mmol/L	2.2–3.9 mmol/L
Gram-stain	Many Gram-negative bacilli are seen	No organism seen	No organism seen	No organism
CSF PCR multiplex	Negative	Human Herpesvirus-6	Human Herpesvirus-6	Negative

CMM, cells per cubic millimeter; CSF, cerebrospinal fluid; RBC, red blood cell; PCR Polymerase Chain Reaction.

## Data Availability

The data supporting this study’s findings are available from the corresponding author upon reasonable request.

## Abbreviations

The following abbreviations are used in this manuscript:

CSF	Cerebrospinal fluid
PICU	Pediatric intensive care unit
CRP	C-reactive protein
MRI	Magnetic resonance imaging
ECG	Electroencephalography
WES	Whole exome sequencing
WGS	Whole genome sequencing
PNP	Purine nucleoside phosphorylase
BTD	Biotinidase
AED	Antiepileptic drug
HIV	Human immunodeficiency virus
HHV-6	Human herpesvirus-6

## References

[1] Hersi K., Gonzalez F.J., Kondamudi N.P. (2024). Meningitis. [Updated 2023 Aug 12]. StatPearls [Internet].

[2] Elouali A., Ouerradi N., Ayad G., Babakhouya A., Rkain M. (2023). *Salmonella* Meningitis in a Young Infant: A Case Report. Cureus.

[3] Wu H.M., Huang W.Y., Lee M.L., Yang A.D., Chaou K.P., Hsieh L.Y. (2011). Clinical features, acute complications, and outcome of *Salmonella* meningitis in children under one year of age in Taiwan. BMC Infect. Dis..

[4] Burrows W. (1959). Textbook of Microbiology.

[5] Alsubaie S., Alrabiaah A. (2020). Clinical characteristics, acute complications, and neurologic outcomes of *Salmonella* meningitis in Saudi infants and children. J. Pediatr. Infect. Dis..

[6] Hudson J.A., Broad J., Martin N.G., Sadarangani M., Galal U., Kelly D.F., Pollard A.J., Kadambari S. (2020). Outcomes beyond hospital discharge in infants and children with viral meningitis: A systematic review. Rev. Med. Virol..

[7] Ansari A., Li S., Abzug M.J., Weinberg A. (2004). Human herpesviruses 6 and 7 and central nervous system infection in children. Emerg. Infect. Dis..

[8] Bozzola E., Krzysztofiak A., Bozzola M., Calcaterra V., Quondamcarlo A., Lancella L., Villani A. (2012). HHV6 meningoencephalitis sequelae in previously healthy children. Infection.

[9] Hazama K., Shiihara T., Tsukagoshi H., Matsushige T., Dowa Y., Watanabe M. (2019). Rhinovirus-associated acute encephalitis/encephalopathy and cerebellitis. Brain Dev..

[10] Hsu M.H., Hsu J.F., Kuo H.C., Lai M.Y., Chiang M.C., Lin Y.J., Huang H.R., Chu S.M., Tsai M.H. (2018). Neurological complications in young infants with acute bacterial meningitis. Front. Neurol..

[11] Ali K.N., Shareef F.O., Abdul Aziz J.M., Najmadden Z.B., Karim A.H. (2024). Infant *Salmonella* enterica Meningitis: A Rare Case Report and Review of Literature. Cureus.

[12] Ahmed T., Ahmed T. (2023). *Salmonella* meningitis in a young child from Pakistan: A case report. J. Med. Case Rep..

[13] Lukas R.V., Mrugala M.M. (2020). Neurologic Complications of Systemic Cancer. Continuum.

[14] Mamishi S., Kamrani L., Mohammadpour M., Yavarian J. (2014). Prevalence of HHV-6 in cerebrospinal fluid of children younger than 2 years of age with febrile convulsion. Iran. J. Microbiol..

[15] Cadotte N., Moore H., Stone B.L., Pershing N.L., Ampofo K., Ou Z., Pavia A.T., Blaschke A.J., Flaherty B., Crandall H. (2024). Prevalence of and Risks for Bacterial Infections in Hospitalized Children with Bronchiolitis. Hosp. Pediatr..

